# *Akkermansia muciniphila* and Its Bioactive Derivatives: Emerging Regulators of Healthy Aging

**DOI:** 10.1111/acel.70702

**Published:** 2026-09-04

**Authors:** Ting Zhang, Jinyuan Niu, Xiaoyu Hu, Zhiang Hu, Yuan Gao, Jialiang Liu, Ning Chen, Yaomin Hu

**Affiliations:** ^1^ Department of Geriatrics Renji Hospital, Shanghai Jiao Tong University School of Medicine Shanghai China; ^2^ Department of Medicine and Therapeutics, Faculty of Medicine The Chinese University of Hong Kong Hong Kong China; ^3^ Shanghai Jiao Tong University School of Medicine Shanghai China

**Keywords:** age‐related diseases, *Akkermansia muciniphila*, gut microbiota, healthy aging, microbiome therapeutics

## Abstract

The global aging trend underscores the urgent need for innovative interventions against aging‐related decline. Accumulating evidence identifies *Akkermansia muciniphila* ( *A. muciniphila* ) as a key gut microbiota regulator of aging, with its depletion associated with age‐related diseases (ARDs), whereas its abundance is enriched in healthy centenarians. This review summarized current evidence linking *A. muciniphila* to aging, examining their causal relationship, the roles of its bioactive derivatives, underlying mechanisms in aging and ARDs, findings from human clinical trials, and challenges in therapeutic translation. Clinically, *A. muciniphila* depletion correlated with aging and various ARDs, while its supplementation effectively ameliorated neurodegenerative disorders, metabolic dysfunction, musculoskeletal decline, intestinal barrier dysfunction, and atherosclerosis. Mechanistically, *A. muciniphila* and its derivatives (Amuc_1100, Amuc_1409, extracellular vesicles, and metabolites such as SCFAs) exerted anti‐aging effects by enhancing intestinal barrier function, maintaining metabolic homeostasis, suppressing chronic inflammation, and modulating immune function, ultimately improving glucolipid metabolism, insulin sensitivity, cognitive function, musculoskeletal health, and vascular health. Emerging clinical trials further demonstrated its translational potential in ameliorating age‐related sarcopenia, metabolic dysfunction, and respiratory symptoms. Despite promising preclinical and clinical results, translational applications reserve challenges related to strain heterogeneity, antimicrobial resistance gene transfer risk, biosafety, production stability, and limited clinical validation in elderly populations. Future research should prioritize large‐scale clinical trials to establish optimal dosage, safety, and long‐term efficacy, while exploring combined microbiota‐targeted therapies. Harnessing the diverse benefits of *A. muciniphila* may enable novel strategies for promoting healthy aging.

## Graphical abstract

The beneficial gut bacterium *Akkermansia muciniphila* (AKK) declines with age but is enriched in healthy centenarians. Supplementation of AKK extends healthspan and alleviates age‐related decline through direct host–microbe interactions and its bioactive derivatives, which collectively enhance gut barrier integrity, restore metabolic homeostasis, suppress chronic inflammation, and modulate immune function.

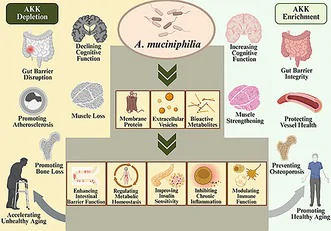

## 1 Introduction

The global population is aging rapidly, making healthy aging a critical social challenge. Healthy aging aims not only to extend lifespan but also to enhance quality of life and mitigate the chronic disease burden. Growing evidence established the gut microbiota as a key regulator of aging (Collado et al. 2024; Sun et al. 2023; Tropini 2024), influencing metabolism, immunity, cognition, and other physiological functions (Aljumaah et al. 2022; Conway and Duggal 2021; Ghosh et al. 2022). Among gut microbes, *Akkermansia muciniphila* ( *A. muciniphila* ) has emerged as a promising modulator of host health, particularly in age‐related decline (Cani et al. 2022). Numerous studies have linked *A. muciniphila* to aging and aging‐related diseases (ARDs) (Zeng et al. 2023).

*A. muciniphila* , a mucin‐degrading bacterium colonizing the intestinal mucus layer (Zhang et al. 2019), contributes to barrier integrity, immune regulation and metabolic homeostasis. It has been implicated in obesity, type 2 diabetes mellitus (T2DM), osteoporosis, neurodegenerative diseases, and cancer (Amorim Neto et al. 2022; Bodogai et al. 2018; Cani et al. 2022; Huang et al. 2025; Liu, Chen, et al. 2021; Rodrigues et al. 2022; L. Wang et al. 2020). *A. muciniphila* abundance decreased with age in the human gut (Collado et al. 2007). Furthermore, oral administration of *A. muciniphila* could elevate systemic metabolites associated with antiaging (Grajeda‐Iglesias et al. 2021). Numerous studies reported that antiaging plant supplements increased *A. muciniphila* abundance to different degrees (Chen et al. 2022; Hong et al. 2022; Liu, Wang, et al. 2021; Yang et al. 2021). However, the causal relationship between *A. muciniphila* and aging, as well as the mechanistic contributions of *A. muciniphila* and its bioactive factors (proteins, extracellular vesicles, and key metabolites), remains unclear.

Several reviews have summarized the association between *A. muciniphila* and aging (Vorontsov et al. 2025; Zeng et al. 2023). Our review extends these by synthesizing clinical trial data, establishing a direct‐versus‐indirect mechanism framework, integrating human genetic evidence, and discussing translational challenges, within a framework distinguishing physiological from disease‐mediated aging. This review examines the correlational and causal roles of *A. muciniphila* in healthy aging and ARDs, elucidates its possible mechanisms, evaluates its therapeutic prospects, and discusses current challenges and future directions.

## 2 Correlation Between *A. muciniphila* and Aging

### 2.1 Human Studies: *A. muciniphila* and Aging

In the context of physiological aging, *A. muciniphila* colonizes the human gut in early infancy and reaches near‐adult levels within 1 year (Collado et al. 2007). As shown in Table 1, its abundance positively correlated with younger biological age and higher physical capability (Tzemah‐Shahar et al. 2024), and declined with age (by > 1 log unit in elderly versus adults, *p* < 0.05) (Collado et al. 2007). However, higher levels of *A. muciniphila* were consistently observed in healthy aging and long‐lived populations (Biagi et al. 2016; Zhang et al. 2024; Ji et al. 2025; Kim et al. 2019; Kong et al. 2016; Liu et al. 2024; Palmas et al. 2022; Singh et al. 2019; Wu et al. 2022), whereas its depletion was associated with unhealthy aging, premature aging, and the transition to end‐of‐life status (Bárcena et al. 2019; Luan et al. 2020; Singh et al. 2019).

**TABLE 1:** Correlation between *A. muciniphila* abundance and aging in humans and animals.

Category	Subject	Key finding	Representative references
Humans			
Centenarians/longevity	Long‐lived vs. younger adults	AKK abundance ↑ in long‐lived individuals	Ji et al. (2025); Chen, Chen, et al. (2024); Chen, Zhang, et al. (2024); Liu et al. (2024); Palmas et al. (2022); Wu et al. (2022); Kim et al. (2019); Biagi et al. (2016); Kong et al. (2016)
Age‐related physiological decline	Elderly vs. adults	AKK abundance ↓ with age	Collado et al. (2007)
Premature aging	HGPS/NGPS patients vs. healthy	AKK abundance ↓	Bárcena et al. (2019)
End‐of‐life transition	Centenarians	AKK ↓ during transition from a healthy state to a terminal state	Luan et al. (2020)
Neurodegenerative diseases	PD patients	AKK ↑ in some PD studies	Zhang et al. (2023)
Cognitive impairment	aMCI vs. normal cognition	AKK ↓ in aMCI	Ma et al. (2025)
Metabolic disorders	T2DM/DCI vs. healthy controls	AKK ↓ in T2DM and DCI	Du et al. (2026)
Musculoskeletal disorders	OP vs. controls	AKK ↓ and positively correlated with BMD	Qin et al. (2021)
Biological age	Younger vs. older biological age	AKK ↑ with younger biological age	Tzemah‐Shahar et al. (2024)
Healthy aging	Healthy vs. non‐healthy aging	AKK ↑ in healthy aging	Singh et al. (2019); Shen et al. (2018)
Animals			
Natural aging	Young vs. aged mice	AKK ↓ with age	Shin et al. (2021); Bodogai et al. (2018); van der Lugt et al. (2018); Fransen et al. (2017); Zhu et al. (2024)
Premature aging	Progeroid mice vs. WT	AKK ↓ in progeroid models	Bárcena et al. (2019)
Aging models	Aged vs. young mice	AKK ↑ with age in certain models	Melo‐Miranda et al. (2025); Gámez‐Macías et al. (2024); Crossland et al. (2023); Zhang et al. (2022)

Abbreviations: AKK, *Akkermansia muciniphila* ; aMCI, amnestic mild cognitive impairment; BMD, bone mass density; DCI, diabetic cognitive impairment; HGPS, Hutchinson‐Gilford progeria syndrome; NGPS, Nestor‐Guillermo progeria syndrome; OP, osteoporosis; PD, Parkinson's disease; T2DM, type 2 diabetes mellitus; WT, wild type.

These seemingly contradictory findings, declining *A. muciniphila* in elderly but enrichment in centenarians, reflect a distinction between chronological aging and healthy longevity. The decline likely resulted from diet, sedentary behavior, chronic health conditions, medication, immunesenescence, and inflammaging, which disrupted gut homeostasis (Conway and Duggal 2021; Franceschi et al. 2018). In contrast, enrichment in long‐lived populations may represent a selective advantage, where preserved *A. muciniphila* supported barrier integrity, attenuated metabolic dysregulation and inflammaging (Cani et al. 2022; Depommier et al. 2019; Ma et al. 2023), and is associated with favorable immune profiles in healthy aging individuals (Shen et al. 2018). Thus, *A. muciniphila* abundance appeared to be a biomarker of biological resilience and healthy aging, not merely chronological age.

### 2.2 Human Studies: *A. muciniphila* and ARDs

**FIGURE 1:**
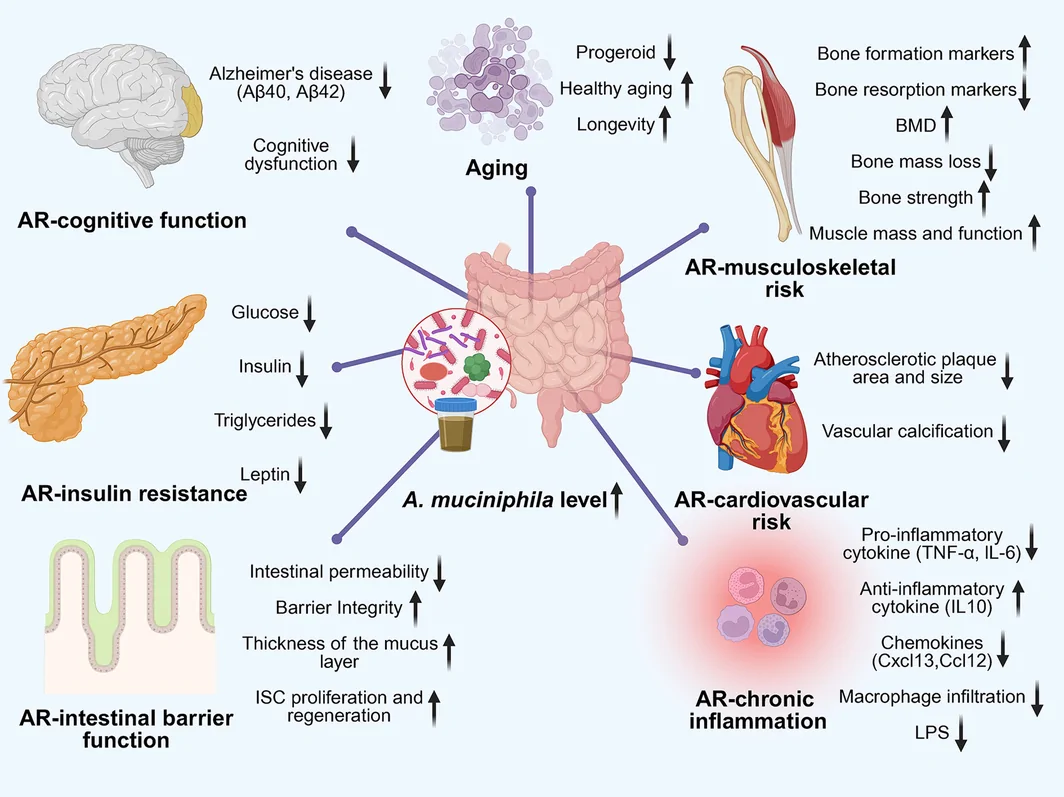
Age‐dependent changes in *A. muciniphila* abundance and its association with ARDs. ARD, aging‐related disease; BMD, bone mineral density; CCL12, C‐C motif chemokine ligand 12; CXCL13, C‐X‐C motif chemokine ligand 13; IL‐6, interleukin‐6; IL‐10, interleukin‐10; ISC, intestinal stem cell; LPS, lipopolysaccharide; TNF‐α, tumor necrosis factor‐α.

Beyond physiological aging, the abundance of *A. muciniphila* has also been linked to disease‐mediated aging phenotypes (Figure 1). Clinically, *A. muciniphila* abundance correlated with several ARDs (Zeng et al. 2023). In osteoporosis, its levels positively associated with bone mineral density and bone formation markers (Keshavarz Azizi Raftar et al. 2021; Qin et al. 2021). However, heterogeneous findings have emerged in neurodegenerative disorders. Some studies reported elevated *A. muciniphila* in Alzheimer's disease (AD) (Khedr et al. 2022; Li et al. 2024; Ubeda et al. 2022; Wang, Li, et al. 2022) and Parkinson's disease (PD) (Hamaguchi et al. 2026; Kleine Bardenhorst et al. 2023; Zhang et al. 2023). Conversely, other studies observed reduced *A. muciniphila* abundance in amnestic mild cognitive impairment (aMCI) (Ma et al. 2025) or AD (Wang et al. 2025). Furthermore, Fan et al. (2025) found that *A. muciniphila* abundance correlated with reduced amyloid burden in MCI and AD patients. Interventional studies also suggested protective effects of *A. muciniphila* in AD‐related models. Oral *A. muciniphila* alleviated cognitive impairment and anxiety‐like behaviors in AD rats (Maftoon et al. 2024), and dietary interventions that increased *A. muciniphila* abundance correlated with improved AD biomarkers in older adults (Nagpal et al. 2019).

These heterogeneous findings may reflect differences in disease stage, observational versus interventional study designs, taxonomic resolution, and host factors. Increased *A. muciniphila* was observed in mild AD (Wang, Li, et al. 2022) and early non‐dementia stages (Ubeda et al. 2022), but decreased in advanced AD (Wang et al. 2025), suggesting stage‐dependent dynamics. The distinction between observational association and causation is also critical. While cross‐sectional studies showed positive correlations between *A. muciniphila* and AD, animal models demonstrated cognitive improvement upon *A. muciniphila* administration (Maftoon et al. 2024), suggesting elevated levels in patients may be compensatory rather than pathogenic. Additionally, most studies used 16S rRNA sequencing, providing mainly genus‐level resolution and may thus obscure species‐specific effects. Finally, host factors such as age, genetics, and diet may modulate *A. muciniphila* abundance and its functional outcomes. A modified Mediterranean‐ketogenic diet was reported to shape the relationship between *A. muciniphila* and neurological health (Nagpal et al. 2019). Collectively, these results highlight a context‐dependent role of *A. muciniphila* in neurodegeneration, warranting further stratified investigations.

### 2.3 Animal Studies

Animal models also supported a role for *A. muciniphila* in aging (Table 1) (Bárcena et al. 2019; Bodogai et al. 2018; Shin et al. 2021; Zhu et al. 2024). Its abundance was reduced in premature aging models (Bárcena et al. 2019) and naturally aged mice (Bodogai et al. 2018; Fransen et al. 2017; Shin et al. 2021; van der Lugt et al. 2018; Zhu et al. 2024), mirroring human findings. However, some studies observed increased abundance in aged mice (Crossland et al. 2023; Gámez‐Macías et al. 2024; Melo‐Miranda et al. 2025; Zhang et al. 2022). These divergent results may reflect differences in mouse strains, housing conditions, diet, or health status. Notably, *A. muciniphila* was consistently depleted in premature aging models, while its abundance in naturally aged mice varied, suggesting that its levels may reflect biological health status rather than serve as a universal chronological age marker. These divergent findings across physiological versus disease‐accelerated aging models highlight the importance of considering aging type when interpreting *A. muciniphila* abundance and designing interventions.

## 3 Causal Relationship Between *A. muciniphila* and Aging

### 3.1 Causal Evidence From Human Genetic Studies

Observational studies and animal models cannot fully exclude confounding or reverse causation. Mendelian randomization (MR), using genetic variants as instrumental variables, has emerged as a powerful approach to strengthen causal inference in human populations (Davey Smith and Hemani 2014). Chen, Chen, et al. (2024) analyzed GWAS summary data and found that genetically predicted *A. muciniphila* abundance was positively associated with parental longevity, including mother's age at death, combined parental age at death, and both parents in the top 10% of survival. In another study using bidirectional MR, Zhang et al. demonstrated that *A. muciniphila* was causally linked to multiple aging‐related traits, including mvAge, appendicular lean mass, and grip strength, suggesting a protective role against age‐related muscle decline and biological aging (Zhang, Lu, et al. 2025). These MR findings provided human genetic evidence supporting a causal contribution of *A. muciniphila* to healthy aging, bridging observational correlations and experimental interventions.

### 3.2 *A. muciniphila* Supplementation in Physiological Aging

As shown in Table 2, preclinical models demonstrated that *A. muciniphila* supplementation extended healthspan in physiological aging models. Fecal microbiota transplantation (FMT) enriched with *A. muciniphila* , or direct administration of *A. muciniphila* , promoted healthy aging and increased lifespan in both naturally aged mice (Cerro et al. 2022). These benefits may be associated with reduced intestinal permeability, restored colonic mucus thickness, attenuated immune dysfunction, alleviated inflammation, improved gut microbiota profiles, and decreased reactive oxygen species (Ma et al. 2023; Van Buiten et al. 2024; van der Lugt et al. 2019). Additionally, pasteurized *A. muciniphila* extended the healthy lifespan in nematodes by reducing glucose, advanced glycation end products, and lipid accumulation (Wu et al. 2025). Amuc_1409, as a secretory protein of *A. muciniphila* , also exhibited protective effects in natural aging mice by enhancing the proliferation and regeneration of intestinal stem cells (ISC) (Kang, Kim, et al. 2024). Collectively, these findings indicate that *A. muciniphila* supplementation promotes healthspan in the context of physiological aging.

**TABLE 2:** Effects of *A. muciniphila* on aging and ARDs in murine models.

Disease model	Subject	AKK status	Treatment	Key outcomes	References
Neurodegenerative disorders					
DCI	HFD‐fed mice	Live/Pasteurized	5 × 10^9^ CFU/day, 200 μL/day, 8 weeks, oral gavage	↑: improved cognitive function, increased synaptic plasticity ↓: reduced neuroinflammation and serum LPS	Du et al. (2026)
AD	APP/PS1 mice	Live	2 × 10^8^ CFU/day, 3 months, oral gavage	**↑**: improve cognitive function **↓**: reduce Aβ plaque deposition, neuroinflammation	Wang et al. (2026)
AD	AD rats	Live	1 × 10^9^ CFU/mL, 0.2 mL/10 g body weight, 5 weeks, oral gavage	**↓**: reduce hippocampal inflammation **↑**: improve cognitive function and anxiety‐like behaviors	Maftoon et al. (2024)
Aging‐related cognitive decline	Aged mice	Amuc_1100	3 μg/200 μL, every 3 days, 6 months, oral gavage	**↑**: improve aging‐related cognitive function via L‐arginine/NO pathway	He et al. (2024)
Cognitive function	Aged mice	Pasteurized	200 μL, twice‐daily, 2 months, oral gavage	**↓**: treatment with AKK enriched by metformin, alleviate inflammatory pathways **↑**: improve cognition	Zhu et al. (2023)
Smoke‐induced cognitive decline	Aged mice	OMVs/ILA	1 μg OMV or 10 mg/kg ILA, every other day for 6 administrations	**↑**: improve cognitive function **↓**: reduce neuroinflammation and synaptic damage	Zhu et al. (2026)
Metabolic disorders					
Obesity	Aged mice	Live	2 × 10^8^ CFU, every other day, 3 weeks, oral gavage	**↑**: improve immune regulation **↓**: reduce age‐related obesity, inflammation	Liu, Chen, et al. (2025)
Insulin resistance	Aged mice	Live	1 × 10^8^ CFU, every second day, 20 days, oral gavage	**↑**: reverse age‐related IR	Bodogai et al. (2018)
Insulin resistance	Mice (selenium‐deficient diet)	Live	1 × 10^9^ CFU (middle‐aged), 2 × 10^8^ CFU (mature), oral gavage	**↑**: improve glucose tolerance and insulin sensitivity **↑**: restore gut barrier integrity and mucus layer thickness **↓**: reduce inflammation	Huang et al. (2025)
Musculoskeletal disorders					
IVDD	Mice	Live/EVs/Protein	1.5 × 10^8^ CFU, 100 μg EVs, or 50 μg B2UKX5, twice weekly, oral gavage	**↑**: improve disc height index **↓**: reduce histological degeneration scores	Guan et al. (2026)
Sarcopenia	SAMP8 mice	Live	1 × 10^9^ CFU, 5 times/week, 3 months, oral gavage	**↑**: enhance grip strength and skeletal muscle mass **↑**: improve gut barrier integrity **↓**: reduce inflammation	Park et al. (2025)
Osteoporosis	OVX mice	Live	Twice weekly, 8 weeks, oral	**↑**: correct bone metabolism imbalance and improve osteoporosis	Liu, Chen, et al. (2021)
Cardiovascular disorders					
Atherosclerosis	*ApoE* ^*−/−*^ mice	LOS	0.2/0.5 mg/kg, every 2 days, 8 weeks, intraperitoneal injection	**↓**: reduce plasma lipids, atherosclerotic lesion area, and plasma LPS **↑**: improve intestinal barrier	Zhang et al. (2026)
Atherosclerosis	*ApoE* ^*−/−*^ mice	Live, inactivated	5 × 10^6^ CFU/day, 8 weeks, oral gavage	**↑**: attenuate lesion area and plaque size of atherosclerosis, restored gut barrier integrity, ameliorated inflammation	Li et al. (2016)
General aging					
Healthspan/lifespan	Natural aging mice	Live	2 × 10^8^ CFU/day, 4 weeks, oral	Improved immunity, reduced inflammation **↑**: improve immunity and oxidative stress **↓**: lower peritoneal pro‐inflammatory cytokines	Cerro et al. (2022)
Healthspan/lifespan	Natural aging mice	Live	3 × 10^9^ CFU, daily (week 1), then every other day, 2–12 weeks, oral gavage	**↑**: improve systemic aging‐related disorders	Ma et al. (2023)
Healthspan	Natural aging mice	—	4.9 × 10^8^ CFU/day, oral gavage	**↑**: attenuate intestinal senescence phenotypes and extend healthspan, improve frailty indices and reverse muscle atrophy	Shin et al. (2021)
Accelerated aging	Ercc1^−/Δ7^ mice	—	2 × 10^8^ CFU, 3 times/week, 10 weeks, oral gavage	**↑**: prevent age‐related mucus thinning, reduce inflammation and immune‐related processes **↑**: promote intestinal health and healthy aging	van der Lugt et al. (2019)
Progeroid phenotype	Lmna^G609G/G609G^ mice	Live	2 × 10^8^ CFU, 3 times/week, starting at 12 weeks of age until death, oral gavage	**↑**: extend average lifespan of progeroid mice	Bárcena et al. (2019)

Abbreviations: ↑, increase or improvement; ↓, decrease or reduction; AD, Alzheimer's Disease; AKK, *Akkermansia muciniphila* ; Aβ, amyloid‐beta; CFU, colony‐forming units; DCI, diabetic cognitive impairment; DHI, disc height index; EVs, extracellular vesicles; HFD, high‐fat diet; ILA, indole‐3‐lactic acid; IR, insulin resistance; IVDD, intervertebral disc degeneration; LOS, lipooligosaccharide; LPS, lipopolysaccharide; OMVs, outer membrane vesicles; OVX, ovariectomy; WT, wild type.

### 3.3 *A. muciniphila* Supplementation in Disease‐Mediated Aging

**FIGURE 2:**
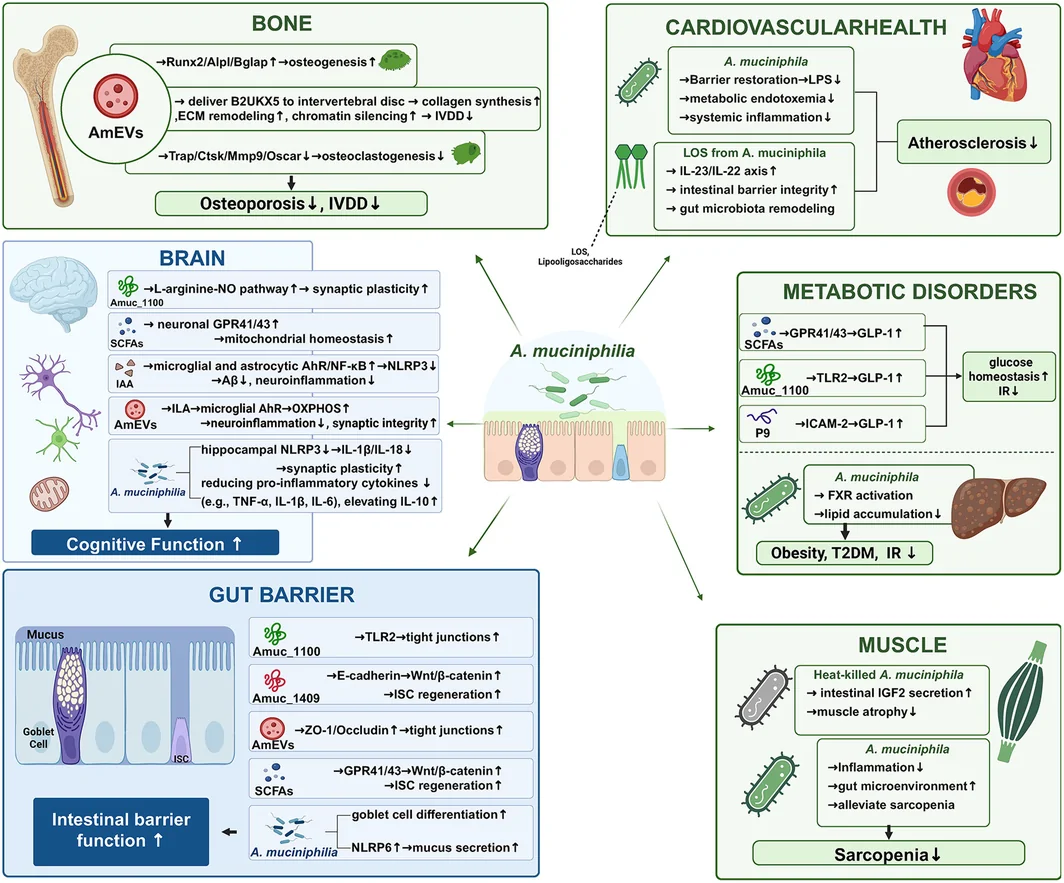
Disease‐centered overview of *A. muciniphila* and its bioactive derivatives in modulating ARDs. The pathways are presented by disease type: Cognitive function, metabolic disorders, muscle, bone, gut barrier, and cardiovascular health. Each panel details the pathways of *A. muciniphila* (membrane proteins, secreted proteins, extracellular vesicles, and metabolites) in each disease context. ARD, aging‐related disease; ILA, indole‐3‐lactic acid; IVDD, intervertebral disc degeneration; LOS, lipooligosaccharides; OXPHOS, oxidative phosphorylation.

As shown in Figure 2, the therapeutic potential of *A. muciniphila* extends across multiple ARDs through disease‐specific mechanisms (Guo et al. 2022). For neurodegenerative disorders, *A. muciniphila* ameliorated cognitive deficits in PD (Fang et al. 2021) and AD (Maftoon et al. 2024), with metformin‐enriched *A. muciniphila* improving cognitive function by reducing IL‐6 (Zhu et al. 2023). Mechanistically, these effects involved Amuc_1100‐mediated synaptic enhancement, SCFA‐dependent mitochondrial homeostasis, and AhR/NLRP3‐mediated neuroinflammation suppression (Guo et al. 2023; He et al. 2024; Wang et al. 2025). Three 2026 studies further showed that *A. muciniphila* inhibited hippocampal NLRP3 inflammasome in diabetic cognitive impairment (Du et al. 2026), reduced Aβ deposition through indole‐3‐acetic acid (IAA) via AhR/NF‐κB/NLRP3 pathway in AD mice (Wang et al. 2026), and delivered indole‐3‐lactic acid via AmEVs to the hippocampus, reprogramming microglial metabolism and suppressing neuroinflammation (Zhu et al. 2026). For metabolic disorders, *A. muciniphila* alleviated age‐related obesity (Liu, Chen, et al. 2025), insulin resistance (IR) (Bodogai et al. 2018) and DM (Rodrigues et al. 2022) in aged mice. *A. muciniphila* improved glycemic control, IR and lipid metabolism, primarily through GLP‐1 upregulation and FXR activation (Jiao et al. 2015; Koh et al. 2016; Liu, Liu, et al. 2025; Mo et al. 2024; Plovier et al. 2017).

In the musculoskeletal system, *A. muciniphila* attenuated muscle atrophy (Shin et al. 2021) and alleviated sarcopenia by inhibiting inflammation (Park et al. 2025), as supported by a clinical trial showing enhanced muscle strength in elderly participants (Kang, Jung, et al. 2024). Mechanistically, heat‐killed *A. muciniphila* increased intestinal IGF2 secretion to prevent muscle atrophy (Zhang et al. 2024). AmEVs further protected against osteoporosis and intervertebral disc degeneration by promoting osteogenesis, inhibiting osteoclastogenesis, and delivering B2UKX5 to intervertebral discs (Guan et al. 2026; Liu, Chen, et al. 2021; Zeng et al. 2023). For cardiovascular health, *A. muciniphila* protected against atherosclerosis by restoring gut barrier function and reducing endotoxemia (Li et al. 2016), while its lipooligosaccharides activated the IL‐23/IL‐22 axis and reshaped gut microbiota composition (Zhang et al. 2026). Notably, intestinal barrier dysfunction serves as a shared pathogenic pathway across multiple ARDs (Kim et al. 2021; Korinek et al. 1998; Pinto et al. 2003; Plovier et al. 2017; Wade et al. 2023; Yu et al. 2023; Zheng et al. 2023). The detailed mechanisms were discussed in Sections 4 and 5. Collectively, *A. muciniphila* emerged as a therapeutic target for physiological aging and ARDs (Table 2).

### 3.4 *A. muciniphila* Supplementation and the Gut Microbial Community

Depommier et al. (2019) found that 3‐month supplementation with live or pasteurized *A. muciniphila* did not alter the overall gut microbiome structure in overweight/obese adults, a finding later confirmed in overweight/obese T2D patients (Zhang, Liu, et al. 2025). Similarly, no community structure shifts were observed in murine atherosclerosis models (Li et al. 2016) or diet‐induced obese mice (Everard et al. 2013). In contrast, SAMP8 mice showed improved α‐diversity and shifted β‐diversity following *A. muciniphila* supplementation (Park et al. 2025), indicating that microbial community modulation may occur in certain pathological contexts. Notably, in vitro co‐culture studies demonstrated cross‐feeding between *A. muciniphila* and butyrate‐producing bacteria including *A. caccae* and *R. species* (Chia et al. 2018; Pichler et al. 2020), suggesting that *A. muciniphila* may exert indirect effects through the promotion of SCFA‐producing commensals. Collectively, while *A. muciniphila* does not drastically remodel the overall gut community, its benefits may be mediated through indirect cross‐feeding with SCFA producers in specific contexts. This distinction between direct and indirect effects was further elaborated in Section 5.

## 4 Antiaging Effects of *A. muciniphila* ‐Derived Bioactive Factors

### 4.1 *A. muciniphila* Effector Proteins

Proteins derived from *A. muciniphila* have emerged as key mediators of its antiaging effects. Amuc_1100, a well‐characterized outer membrane protein, functioned primarily through TLR2 activation, ameliorating age‐related conditions such as intestinal barrier dysfunction and IR (Cani and de Vos 2017; Plovier et al. 2017). Amuc_1100 also correlated with the attenuation of NF‐κB‐mediated inflammation (Qu et al. 2023; Wang, Jin, et al. 2024). Additionally, this protein could enhance cognitive function in aged mice by increasing L‐arginine, nitric oxide and endothelial nitric oxide synthase in the brain (He et al. 2024). Amuc_1409 (Kang, Kim, et al. 2024), another significant secreted protein, ameliorated age‐related decline in intestinal regenerative capacity. It counteracted the functional deterioration of ISCs by activating the Wnt/β‐catenin signaling pathway, effectively restoring ISC populations and supporting epithelial maintenance during aging (Kang, Kim, et al. 2024).

### 4.2 *A. muciniphila* ‐Derived Extracellular Vesicles

*A. muciniphila* ‐derived extracellular vesicles (AmEVs) are nano‐sized particles carrying proteins, lipids, nucleic acids, and other active components (Ahmadi Badi et al. 2017). They regulate intestinal barrier function by upregulating ZO‐1 and MUC2, and suppress pro‐inflammatory cytokines including TNF‐α, IL‐1β, and IL‐6 (Zheng et al. 2023). Recent studies have extended their role to bone health. AmEVs can translocate to bone tissues and modulate bone metabolism by promoting osteogenesis through upregulation of Runx2, Alpl and Bglap, and inhibiting osteoclastogenesis through downregulation of Trap, Ctsk, Mmp9, Oc‐stamp, and Oscar (Liu, Chen, et al. 2021). Similarly, AmEVs delivered the effector protein B2UKX5 to intervertebral discs, promoting collagen synthesis and extracellular matrix remodeling to attenuate disc degeneration (Guan et al. 2026). These findings position AmEVs as a potential nanotherapeutic candidate for preventing age‐related bone loss, particularly in the context of postmenopausal osteoporosis. Recent evidence further showed that AmEVs delivered neuroprotective metabolites to the brain, reprogramming microglial metabolism and suppressing neuroinflammation through AhR signaling (Zhu et al. 2026).

### 4.3 Short‐Chain Fatty Acids

Short‐chain fatty acids (SCFAs), such as acetate and propionate, are key metabolites of *A. muciniphila* that maintain intestinal barrier integrity (Macia et al. 2012) and exert anti‐inflammatory effects through G protein‐coupled receptors (Koh et al. 2016; Mann et al. 2024; Venegas et al. 2019). Mechanistically, SCFAs directly promoted goblet cell differentiation and mucus production through GPR41 (Wang, Cai, et al. 2024), and indirectly modulated immune cells (such as macrophages and T cells) through GPR43 and GPR109A, thereby creating an anti‐inflammatory environment that supports intestinal barrier function (Mann et al. 2024; Venegas et al. 2019). These SCFAs contributed to *A. muciniphila* 's antiaging effects by reducing pulmonary inflammaging, oxidative stress, and metabolic dysregulation in old mice (Hildebrand et al. 2023). Venegas et al. elucidated that acetate extended lifespan in *Caenorhabditis elegans* and protected against aging in naturally aging mice (Venegas et al. 2019). Reduced butyrate promoted IR, while butyrate supplementation restored insulin responses in aged mice and rhesus macaques (Bodogai et al. 2018). Notably, SCFAs (primarily propionate) derived from *A. muciniphila* can cross the blood–brain barrier and activate neuronal GPR41/GPR43 signaling, thereby maintaining mitochondrial homeostasis and ameliorating AD pathology (Wang et al. 2025).

## 5 Mechanisms of *A. muciniphila* in Modulating Aging and ARDs

Within the “Hallmarks of Aging” framework (López‐Otín et al. 2013, 2023), this discussion examined how *A. muciniphila* modulated key aging hallmarks. Mechanisms were distinguished as direct host–microbe interactions versus indirect microbiota‐mediated effects. Direct effects involved binding of *A. muciniphila* or its products (e.g., Amuc_1100 and Amuc_1409) to host receptors (TLR2 and E‐cadherin) (Kang, Kim, et al. 2024; Plovier et al. 2017). Amuc_1100 correlated with the attenuation of NF‐κB‐mediated inflammation (Qu et al. 2023; Wang, Jin, et al. 2024), while Amuc_1409 activated Wnt/β‐catenin signaling to enhance ISC regeneration (Kang, Kim, et al. 2024).

Indirect effects were mediated by bacterial metabolites such as SCFAs, which promoted goblet cell function via GPR41 and modulated immune cells via GPR43/GPR109A (Mann et al. 2024; Venegas et al. 2019; Wang, Cai, et al. 2024). SCFAs also acted on L cells GPR41/43 to promote GLP‐1 release (Koh et al. 2016) or shaped the gut ecosystem through cross‐feeding networks (e.g., with butyrate‐producing bacteria like *Anaerostipes caccae* ) (Chia et al. 2018). Indirect effects also included cell‐autonomous responses in goblet cells, such as NLRP6 inflammasome activation, which can be restored by *A. muciniphila* colonization to promote mucus secretion and enhance autophagy (Yu et al. 2023). Notably, Amuc_1100 and Amuc_1409 converged on barrier reinforcement but acted through distinct receptors (TLR2 vs. E‐cadherin) and have not yet been examined for synergy.

### 5.1 Enhancing Intestinal Barrier Integrity

**FIGURE 3:**
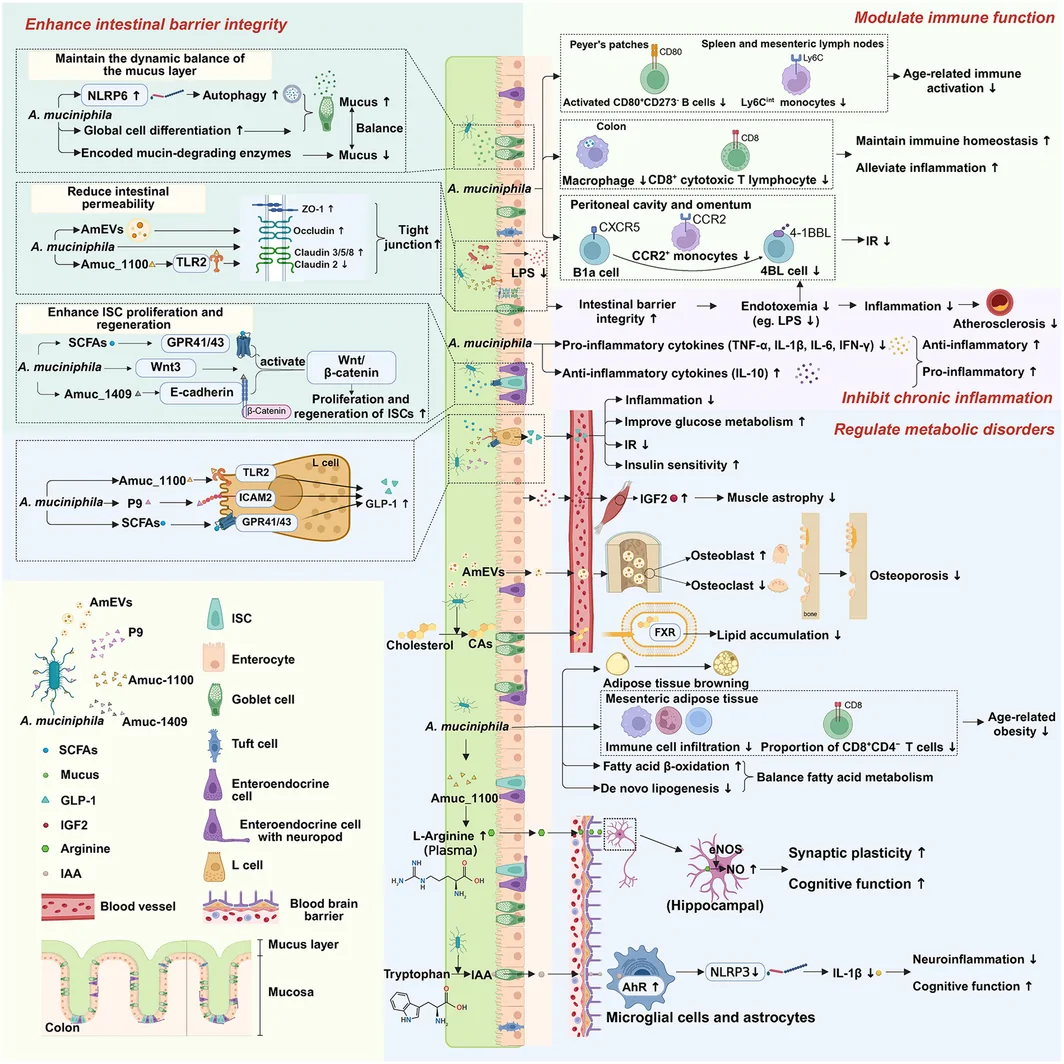
Potential mechanisms of *A. muciniphila* and its derived factors in modulating aging and ARDs. *A. muciniphila* exerted antiaging effects through direct host–microbe interactions (e.g., Amuc_1100‐TLR2 and Amuc_1409‐E‐cadherin) and indirect microbiota‐mediated effects (e.g., SCFAs), which acted through pathways including intestinal barrier enhancement, metabolic regulation, inflammation suppression and immune modulation. ARD, aging‐related disease; AhR, aryl hydrocarbon receptor; CA, cholic acid; eNOS, endothelial nitric oxide synthase; EVs, extracellular vesicles; FXR, farnesoid X receptor; GLP‐1, glucagon‐like peptide‐1; GPR41/43, G‐protein‐coupled receptor 41/43; IAA, indole‐3‐acetic acid; ICAM2, intercellular adhesion molecule 2; IFN‐γ, interferon‐γ; IGF2, insulin‐like growth factor 2; IL‐1β, interleukin‐1β; IR, insulin resistance; ISC, intestinal stem cell; LPS, lipopolysaccharide; NLRP3, NOD‐like receptor thermal protein domain associated protein 3; NO, nitric oxide; P9, protein 9; TLR2, toll‐like receptor 2; TNF‐α, tumor necrosis factor‐α; ZO‐1, zonula occludens‐1.

Aging is associated with reduced colonic mucus thickness, increased intestinal permeability and declined ISC regenerative capacity (Elderman et al. 2017; Nalapareddy et al. 2017; Tran and Greenwood‐Van Meerveld 2013). *A. muciniphila* ameliorated these senescence‐related phenotype in aged mice and extended healthspan (Shin et al. 2021). As shown in Figure 3, *A. muciniphila* improved intestinal barrier function through enhanced mucus production, reduced intestinal permeability, and ISC regeneration (Mo et al. 2024). By preserving ISC function and maintaining barrier integrity, *A. muciniphila* contributes to counteracting the aging hallmark of stem cell exhaustion.

First, *A. muciniphila* restores the intestinal mucus layer. Long‐term supplementation restored age‐related mucus thickness decline (van der Lugt et al. 2019). Specifically, it promoted goblet cell differentiation via Wnt/β‐catenin signaling (Kim et al. 2021; Korinek et al. 1998; Pinto et al. 2003) and enhanced mucus secretion through restoration of NLRP6 inflammasome expression and autophagy (Yu et al. 2023). Additionally, *A. muciniphila* also encoded mucin‐degrading enzymes, including glycosidases, sialidases, sulfatases, and proteases (Derrien et al. 2010; Everard et al. 2013; Sicard et al. 2017), stimulating dynamic mucus renewal. Second, *A. muciniphila* reduces intestinal permeability and reinforces tight junctions. It upregulated tight junction proteins, including ZO‐1, Occludin, and Claudins (Li et al. 2016). This effect is also mediated by Amuc‐1100 through TLR2 signaling (Plovier et al. 2017; Wade et al. 2023), as well as by AmEVs (Chelakkot et al. 2018; Wang et al. 2023). Third, *A. muciniphila* promotes epithelial regeneration by ISC activation. It promoted ISC proliferation through Wnt/β‐catenin signaling, partly mediated by GPR41/43 receptors (Kim et al. 2021). The secreted protein Amuc_1409 directly binded to E‐cadherin, facilitating β‐catenin dissociation, and activating Wnt/β‐catenin signaling to enhance ISC regeneration in intestinal organoids and aging mouse models (Kang, Kim, et al. 2024). This ISC‐supporting effect counteracts stem cell exhaustion, a hallmark of aging (López‐Otín et al. 2013).

The therapeutic potential of *A. muciniphila* extends to other age‐related conditions. Amuc_1100 restored plasma L‐arginine and hippocampal nitric oxide levels, thereby improving synaptic plasticity and alleviating cognitive decline in aged mice (He et al. 2024). In the musculoskeletal system, heat‐killed *A. muciniphila* increased intestinal IGF2 secretion and prevented muscle atrophy (Zhang et al. 2024). Clinically, *A. muciniphila* was more abundant in healthy elderly, inversely correlating with bone resorption markers and positively with bone formation markers and 25‐OH‐D3 (Qin et al. 2021).

### 5.2 Regulating Metabolic Disorders

Aging is associated with metabolic dysfunction, and *A. muciniphila* exerted beneficial effects on glycolipid metabolism and IR. In aged mice, it reduced age‐related obesity (Liu, Chen, et al. 2025). It regulated fatty acid metabolism by activating β‐oxidation while inhibiting de novo lipogenesis, improving glycolipid metabolism and healthy lifespan in *C. elegans* (Wu et al. 2025). Increased *A. muciniphila* abundance also correlated with enhanced fatty acid oxidation and adipose browning, while its depletion linked to increased lipogenesis and IR (Cani et al. 2022; Schneeberger et al. 2015). Supplementation with *A. muciniphila* or enrofloxacin (an antibiotic that increases its abundance) restored insulin sensitivity in aged mice and macaques (Bodogai et al. 2018), and counteracted metabolic endotoxemia and IR in humans with metabolic syndrome (Depommier et al. 2021; Everard et al. 2013). *A. muciniphila* targets the aging hallmark of deregulated nutrient sensing.

A key mechanism is glucagon‐like peptide 1 (GLP‐1) upregulation. Reduced GLP‐1 contributed to DM pathology by impairing glucose and lipid metabolism (Faerch et al. 2015). *A. muciniphila* ‐derived SCFAs improved glycemic control by stimulating GLP‐1 secretion (Cani et al. 2022; Koh et al. 2016). Additionally, Amuc_1100 and protein 9 (P9; encoded by the Amuc_1831 gene (Yoon et al. 2021)) bind TLR2 and ICAM‐2, respectively, on intestinal L cells to promote GLP‐1 secretion (Mo et al. 2024) and regulate glucose homeostasis. Beyond glucose regulation, *A. muciniphila* modulated lipid metabolism by promoting the conversion of cholesterol to cholic acid, activating farnesoid X receptor (FXR) in adipose tissue to suppress lipid accumulation (Jiao et al. 2015; Liu, Liu, et al. 2025).

### 5.3 Inhibiting Inflammation

Age‐related low‐grade chronic inflammation, called inflammageing (Franceschi et al. 2018), promotes cell senescence, autophagy dysfunction, inflammasome activation, and DNA damage (Singh et al. 2024; Tran and Greenwood‐Van Meerveld 2013). Inflammaging is recognized as a central hallmark of aging that links physiological aging and disease‐mediated aging, as it both accelerates natural age‐related decline and drives the pathogenesis of ARDs. Systemically, *A. muciniphila* supplementation decreased levels of inflammatory cytokines such as TNF‐α and IL‐1β in naturally aging mice, enhanced hepatic antioxidant gene expression, and upregulated immune markers in the spleen (Ma et al. 2023). It also downregulated IL‐8 (Pan et al. 2022). Additionally, as discussed in Section 5.1, the barrier restoration by *A. muciniphila* reduced systemic endotoxin (e.g., LPS) translocation, thereby alleviating chronic inflammation. Furthermore, *A. muciniphila* protected against atherosclerotic lesions in Apoe^−^/^−^ mice by inhibiting metabolic endotoxemia‐induced inflammation (Li et al. 2016). These effects were partly mediated by GLP‐1 release (Mo et al. 2024), a hormone with established anti‐inflammatory roles (Mehdi et al. 2023). *A. muciniphila* also counteracted high‐fat diet‐induced adipose inflammation in metabolic syndrome (Everard et al. 2013).

In the central nervous system, *A. muciniphila* alleviated neuroinflammation and cognitive decline (Maftoon et al. 2024; Zhu et al. 2023), reducing pro‐inflammatory cytokines in the hippocampus of AD rats (Maftoon et al. 2024) and elevating IL‐10 (Luo et al. 2025). It also modulated tryptophan metabolism to increase IAA, which activated microglial AhR and inhibited NLRP3 inflammasome activation in AD mice (Guo et al. 2023). Collectively, these findings demonstrated that *A. muciniphila* exerted broad anti‐inflammatory effects across multiple tissues, counteracting the inflammaging hallmark of aging.

### 5.4 Modulating Immune Function

Aging is associated with a progressive dysregulation of the immune system. This phenomenon, termed immunosenescence, involves thymic involution and impaired responses to novel antigens (López‐Otín et al. 2023). *A. muciniphila* counteracts immunosenescence. In aged mice, *A. muciniphila* reduced immune cell infiltration in mesenteric adipose tissue, including a decrease in CD45^+^ total immune cells and CD8^+^ T cells, reducing age‐related obesity (Liu, Chen, et al. 2025). It enhanced chemotaxis, phagocytosis, NK cell activity, and lymphoproliferation while reducing pro‐inflammatory cytokines from peritoneal immune cells, suggesting healthy longevity benefits (Cerro et al. 2022). Moreover, *A. muciniphila* reduced infiltration of pro‐inflammatory macrophages and cytotoxic T lymphocytes, thereby modulating immune responses (Wang et al. 2020). *A. muciniphila* also decreased activated CD80^+^CD273^−^B cells in Peyer's patches and Ly6C^int^monocyte in spleen and mesenteric lymph nodes of accelerated aging mice, attenuating age‐related immune activation at old age (van der Lugt et al. 2019).

Mechanistically, *A. muciniphila* suppressed NLRP3 inflammasome activation, a key driver of age‐related thymic atrophy (Youm et al. 2012). Deletion of NLRP3 enhanced cortical thymic epithelial cell maintenance, expanded T‐cell progenitors, and preserved T‐cell receptor diversity (Youm et al. 2012). Notably, loss of *A. muciniphila* can lead to endotoxin leakage, activating CCR2^+^ monocytes. Upon infiltrating the omentum, these CCR2^+^ monocytes converted B1a cells into 4BL cells, which induced IR through 4‐1BBL expression (Bodogai et al. 2018). Restoration of *A. muciniphila* may normalize immune‐metabolic crosstalk during aging. Collectively, *A. muciniphila* ameliorated aging and ARDs through metabolite‐, effector protein‐, and EV‐mediated interactions that enhanced intestinal barrier integrity, promoted metabolic health, regulated immune homeostasis, and suppressed inflammation, thereby counteracting multiple hallmarks of aging (Figure 3).

## 6 Translating Promise Into Practice: Clinical Trials on *A. muciniphila* and Healthy Aging

Recent clinical trials provided promising evidence for *A. muciniphila* supplementation in modulating age‐related decline (Table 3). Earlier studies established its safety and potential to improve metabolic parameters in overweight/obese individuals (Depommier et al. 2021; Depommier et al. 2019; Plovier et al. 2017). More recent investigations target aging‐related phenotypes. A 12‐week intervention with pasteurized *A. muciniphila* (HB05P) significantly improved lower‐limb muscle strength and increased serum follistatin in adults aged 60 and older, suggesting benefit against sarcopenia (Kang, Jung, et al. 2024). Its heat‐killed form alleviated respiratory symptoms in adults (Lee et al. 2024), potentially ameliorating age‐associated respiratory decline. Two 2026 trials provided additional insights. Suenaert et al. (2026) confirmed baseline‐dependent efficacy of pasteurized *A. muciniphila* in metabolic syndrome, while Wu et al. demonstrated prebiotic‐induced endogenous enrichment of *A. muciniphila* (Wu et al. 2026). The efficacy may depend on formulation and individual baseline microbiota status, as pasteurized forms often demonstrated more consistent metabolic benefits (Aalipanah et al. 2025; Depommier et al. 2021; Depommier et al. 2019), and effects in T2DM were pronounced primarily in individuals with low baseline *A. muciniphila* abundance (Zhang, Liu, et al. 2025). Additionally, a multi‐strain formulation containing *A. muciniphila* improved postprandial glucose control in a T2DM randomized trial (Perraudeau et al. 2020). Collectively, these trials highlight *A. muciniphila* as a modulator of metabolic health and a potential therapeutic candidate for improving physical function and resilience in aging population.

**TABLE 3:** Effects of *A. muciniphila* supplementation in human clinical trials.

Author	Design	Participants	AKK form	AKK intervention	Treatment outcome
Suenaert et al. (2026)	DB, PC, RCT, MulticenterPhase 2 trial (16 weeks)	Overweight or obese adults with metabolic syndrome	Pasteurized AKK	AKK Group: 1 capsule/day of pasteurized AKK Muc^T^ (3 × 10^10^ TFU/capsule) for 16 weeks. Placebo Group: identical capsule (maltodextrin only).	Safety: Well‐tolerated. No serious adverse events. Supplementation with AKK: No differences in Matsuda index, HOMA‐IR, HbA1c, fasting glucose or WC. In participants aged ≥ 63 years: ↑: HOMA‐S In postmenopausal women: ↑: Matsuda index
Zhang, Liu, et al. (2025)	DB, PC, RCT, Multicenter Phase 2 trial (12 weeks)	Overweight or obese adults with T2DM	Live Akk strain AKK‐WST01	AKK‐WST01 Group: 3 sachets/day of live AKK‐WST01 powder (1 g/sachet, 1–5 × 10^10^ CFU/g), totaling 3–15 × 10^10^ CFU/day for 12 weeks. Placebo Group: 3 sachets/day of identical excipient‐only powder.	Safety: Well‐tolerated. No serious adverse events. Supplementation with AKK: No significant differences in body weight, HbA1c or other metabolic parameters were found. In participants with low baseline AKK: ↑: Successful colonization, fat oxidation. ↓: Body weight, fat mass, HbA1c, LDL‐C.
Aalipanah et al. (2025)	DB, PC, RCT	Overweight or obese adults	Heat‐inactivated form	AMY: yogurt fortified with 10^10^ TFU of heat‐inactivated AKK. LRY: yogurt with an equivalent dose of heat‐inactivated LR. The LFY: unfortified low‐fat yogurt. 8 weeks.	Safety: No adverse effects. Supplementation with AKK yogurt: ↓: WC, waist‐to‐height ratio, body fat percentage, aspartate aminotransferase and appetite scores.
Lee et al. (2024)	DB, PC, RCT, Multicenter	Adults with respiratory symptoms for 4–12 weeks	Heat‐killed form, strain EB‐AMDK19	ETB‐F01: capsules containing 5.0 × 10^10^ cells of heat‐killed AKK strain EB‐AMDK19. Placebo: corn starch and maltodextrin.	Safety: Well‐tolerated. No serious adverse events were observed. Supplementation with AKK: ↓: BCSS total score, BCSS breathlessness score and BCSS cough score.
Kang, Jung, et al. (2024)	DB, PC, RCT	Aged 60 years and older	Pasteurized postbiotic form, strain HB05	Treatment: a daily dose of pasteurized AKK strain HB05, containing 1 × 10^10^ cells/day. Placebo: microcrystalline cellulose.	Safety: Well‐tolerated. No serious adverse events. Supplementation with AKK: ↑: Peak torque of left leg extensor muscles, peak torque/body weight of left leg extensor muscles, serum follistatin levels.
Depommier et al. (2021)	DB, PC, RCT	Overweight/obese, IR volunteers with prediabetes/metabolic syndrome	Pasteurized AKK Alive AKK	Placebo: sterile solution of PBS‐containing glycerol. Pasteurized AKK: 10^10^ pasteurized AKK bacteria per day. Alive AKK: 10^10^ live bacteria per day.	Safety: The intervention was safe and well‐tolerated. Pasteurized AKK group: ↓: IR and inflammatory markers. ↓: Liver enzyme levels (AST, ALT, GGT). ↓: Endotoxemia. Alive AKK group: Showed a weaker overall metabolic improvement trend compared to the pasteurized form.
Perraudeau et al. (2020)	DB, PC, RCT, Multicenter (12 weeks)	Adults with T2DM	Live strain	WBF‐011 contained AKK, four other butyrate‐producing strains, and inulin. WBF‐010 was a simplified version without AKK (containing 3 other strains and inulin).	Safety: The intervention was safe and well‐tolerated. WBF‐011 supplementation (containing AKK): ↓: Postprandial glucose (Total AUC, *p* = 0.050; Incremental AUC, *p* = 0.007). ↓: Trend for improved long‐term glucose control (HbA1c, *p* = 0.054).
Depommier et al. (2019)	DB, PC, RCT (single‐center, 3 months)	Overweight/obese, insulin‐resistant volunteers with metabolic syndrome	Pasteurized AKK: Heat‐inactivated (70°C, 30 min) Viable AKK: Live bacteria	Placebo: glycerol in PBS Pasteurized AKK: 10^10^ pasteurized AKK bacteria/day suspended in the same vehicle. Viable AKK: 10^10^ alive bacteria/day suspended in the same vehicle.	Safety & Tolerability: Well‐tolerated with > 99% compliance. No serious adverse events. Pasteurized AKK supplementation: ↑: Improved insulin sensitivity. ↓: Reduced insulinemia and total cholesterol. ↓: Lowered hepatic enzymes (γGT, AST) ↓: plasma LPS levels, WBC count. Viable AKK group: Weaker metabolic improvement trends, not significant vs. placebo.
Plovier et al. (2017)	DB, PC, RCT	Overweight/obese volunteers with metabolic syndrome (*N* = 20):	Live AKK strain Muc^T^ Heat‐inactivated form	Placebo: received the vehicle (glycerol in PBS). Treatment groups: received either 10^9^ CFU viable, 10^10^ CFU viable, or 10^10^ CFU pasteurized AKK bacteria daily.	Safety & Tolerability: No significant changes in markers of inflammation, hematology, kidney, liver or muscle function after 2 weeks. Conclusion: Short‐term administration of both viable and pasteurized AKK were safe and well‐tolerated in overweight/obese individuals.

Abbreviations: ↑, increase or improvement; ↓, decrease or reduction; AKK, *Akkermansia muciniphila* ; AKK, *Akkermansia muciniphila* ; AMY, AKK postbiotic yogurt; BCSS, Breathlessness, Cough, and Sputum Scale; CFU, colony‐forming units; DB, double‐blind; HOMA‐IR, homeostatic modela assessment of insulin resistance; HOMA‐S, homeostatic model assessment of insulin sensitivity; LFY, Low‐fat yogurt; LR, *Lacticaseibacillus rhamnosus*; LRY, *L. rhamnosus* postbiotic yogurt; PC, placebo‐controlled; RCT, randomized controlled trial; TFU, thermal forming units; WBF, whole biome formulation; WC, waist circumference.

## 7 Challenges and Safety Considerations for Clinical Translation

### 7.1 Key Challenges

Several challenges should be addressed to advance its clinical translation. First, strain heterogeneity and functional validation remain primary concerns. Distinct phylogroups and strains exhibited diverse traits, including variations in oxygen tolerance, adhesion, and sulfur acquisition (Becken et al. 2021; Guo et al. 2017). These differences lead to differential host effects, such as strain‐specific anti‐inflammatory efficacy (Zhai et al. 2019). Rigorous preclinical functional validation is essential for identifying safe and effective candidates. Second, antimicrobial resistance gene (ARG) transfer potential poses a critical biosafety risk. ARGs identified in some *A. muciniphila* genomes, located on mobile genetic elements, raised concerns about horizontal transfer to pathogens under antibiotic selection pressure (de Nies et al. 2022; Guo et al. 2017). Comprehensive genomic screening for ARGs is essential, especially for vulnerable aging populations.

Third, the impact of *A. muciniphila* is highly context‐dependent. In the absence of dietary fiber, *A. muciniphila* expanded and upregulated mucin‐degrading enzymes, disrupting the mucus layer, increasing permeability, and exacerbating colitis in mice (Desai et al. 2016). Supplementation without dietary context could be detrimental, particularly when the mucus barrier is compromised. Fourth, aging‐related vulnerabilities pose additional challenges. Elderly individuals exhibited immunosenescence, increased intestinal permeability, polypharmacy and comorbidities (Franceschi et al. 2018; Tran and Greenwood‐Van Meerveld 2013). These factors may affect the safety and efficacy of *A. muciniphila* supplementation. Immunosenescence may alter responses to immunostimulatory components (e.g., Amuc_1100), while polypharmacy could select resistant strains. Dedicated geriatric trials are urgently needed. Finally, production and formulation require optimization for scalable and stable manufacturing. *A. muciniphila* is a strict anaerobe with temperature‐dependent oxygen tolerance (Machado et al. 2020; Ouwerkerk et al. 2016). While pasteurized bacteria or purified derivatives offer more stable alternatives, their long‐term safety and efficacy require further validation. Large‐scale human trials in elderly cohorts are needed to define optimal dosing, assess long‐term efficacy, and explore potential synergies with prebiotics or microbial consortia.

### 7.2 Safety Considerations for Anti‐Aging Interventions

The relationship between *A. muciniphila* and cancer poses a critical safety concern. A recent study (Khalili et al. 2026) reported that *A. muciniphila* and its derivatives reduced tumor burden across multiple cancer types in preclinical models, primarily through CD8^+^ T cell activation, M1 macrophage polarization, and inhibition of the kynurenine/AhR pathway. Some human studies showed reduced abundance of *A. muciniphila* in colorectal adenomas, colorectal cancer (CRC), and ovarian cancer compared to their respective healthy or benign controls (Fan et al. 2021; Wang et al. 2020; Wang, Qin, et al. 2022). However, *A. muciniphila* mono‐colonization increased tumor burden in Apc‐mutant mice (Dingemanse et al. 2015) and exacerbated tumorigenesis in inflammation‐driven CRC (Wang, Cai, et al. 2022). Consistently, several studies reported elevated *A. muciniphila* abundance in patients with CRC (Li et al. 2023; Osman et al. 2021). Given this context‐dependent safety profile, elderly individuals with cancer or precancerous lesions may need exclusion from antiaging interventions.

A personalized approach requires a multi‐dimensional biomarker framework, including baseline fecal *A. muciniphila* abundance for patient stratification (Mount et al. 2026; Zhang, Liu, et al. 2025), serum inflammatory and barrier integrity markers (such as LPS and TNF‐α) to identify responders (Li et al. 2016), baseline cancer screening for safety and strain‐level functional genotyping to match aging phenotypes. Future prospective trials are warranted to validate these candidate biomarkers.

### 7.3 Future Directions: Stratification by Physiological Versus Disease‐Mediated Aging

Although our review distinguishes physiological from disease‐mediated aging, most studies lack stratification by aging type. Human cohorts report associations with chronological age or disease status, not natural versus disease‐accelerated decline. Similarly, preclinical models rarely compare naturally aged and disease‐accelerated models directly. This prevents determining whether *A. muciniphila* effects differ across aging types. Future studies should compare effects across models and stratify human cohorts by age and disease status.

## 8 Conclusions

Accumulating evidence underscores *A. muciniphila* as a promising microbial candidate for promoting healthy aging and mitigating ARDs. Its decline marked unhealthy aging, while restoring its levels, through direct supplementation or its bioactive factors, provides broad protection against ARDs. *A. muciniphila* exerted these benefits both through its direct colonization effects and via its bioactive derivatives, including Amuc_1100, Amuc_1409, AmEVs and metabolites such as SCFAs, which collectively reinforced the intestinal barrier, restored metabolic and immune homeostasis and suppressed chronic inflammation. The trajectory from correlation to causation is now supported by MR studies, animal interventions, and preliminary human trials.

Translating these findings into clinical applications faces significant challenges. Given the distinction between physiological and disease‐mediated aging, future research should investigate whether *A. muciniphila* ‐based interventions act through shared or distinct mechanisms in these two contexts. Distinguishing the causal drivers of *A. muciniphila* decline, whether immunosenescence, inflammaging, dietary shifts, or polypharmacy, requires longitudinal human cohorts with repeated sampling to establish temporal precedence. Endogenous enrichment through microbiota‐accessible substrates (polyphenols, inulin‐type fructans) offers an alternative to exogenous supplementation, but overcoming age‐related barriers may require encapsulated formulations and individualized dosing. The interdependency of gut microbial networks, exemplified by cross‐feeding between *A. muciniphila* and butyrate‐producing bacteria, suggests that single‐strain supplementation may be insufficient and that rationally designed consortia are needed to target multiple aging hallmarks. Addressing these challenges will accelerate the translation of *A. muciniphila* ‐based strategies into clinically viable interventions for healthy longevity.

## Author Contributions

T.Z. and Y.H. contributed to the study design and conception. All authors contributed to the writing, revising, and final approval of the manuscript.

## Funding

This study was funded by the National Natural Science Foundation of China under Grant (number 82300622); Shanghai Sailing Program under Grant (number 23YF1423100); and National Natural Science Foundation of China under Grant (number U22A20287).

## Conflicts of Interest

The authors declare no conflicts of interest.

## Data Availability

Data sharing not applicable to this article as no datasets were generated or analyzed during the current study.
